# Prognosis of a Wind Turbine Gearbox Bearing Using Supervised Machine Learning

**DOI:** 10.3390/s19143092

**Published:** 2019-07-12

**Authors:** Faris Elasha, Suliman Shanbr, Xiaochuan Li, David Mba

**Affiliations:** 1 Faculty of Engineering, Environment & Computing, Coventry University, Coventry CV1 5FB, UK; 2 School of Water, Energy and Environment, Cranfield University, Bedfordshire MK43 0AL, UK; 3 Faculty of Computing, Engineering and Media, De Montfort University, Leicester LE1 9BH, UK; 4 Department of Mechanical Engineering, University of Nigeria, Nsukka 410001, Nigeria

**Keywords:** wind turbine, vibration measurement, regression, artificial neural network, high-speed shaft bearing, prognosis, remaining useful life

## Abstract

Deployment of large-scale wind turbines requires sophisticated operation and maintenance strategies to ensure the devices are safe, profitable and cost-effective. Prognostics aims to predict the remaining useful life (RUL) of physical systems based on condition measurements. Analyzing condition monitoring data, implementing diagnostic techniques and using machinery prognostic algorithms will bring about accurate estimation of the remaining life and possible failures that may occur. This paper proposes to combine two supervised machine learning techniques, namely, regression model and multilayer artificial neural network model, to predict the RUL of an operational wind turbine gearbox using vibration measurements. Root Mean Square (RMS), Kurtosis (KU) and Energy Index (EI) were analysed to define the bearing failure stages. The proposed methodology was evaluated through a case study involving vibration measurements of a high-speed shaft bearing used in a wind turbine gearbox.

## 1. Introduction

The continuous monitoring of wind turbine systems and their constituent components (e.g., drive trains, generators and blades) can be the most effective way to eliminate unplanned maintenance and increase availability. With advanced data acquisition technology and signal processing techniques, faults can be diagnosied in the early stage and suitable maintenance actions such as replacement and repair can be scheduled to prevent the damage from propagating to surrounding areas. Wind turbine systems are operating under adverse condition, such as vastly varying speeds, loads and temperatures. Bearings in wind turbine systems generally operate under adverse conditions such as chemical effects of lubricant, contamination and moisture, as a result, bearings are subject to performance degradation if no preventive actions are taken. In addition, the high demands for renewable energy resources has resulted in further demands on wind turbines availability and reliability, especially on the key components such as the gearbox and bearings [1]. 

A gearbox is one of the most important units in the drive train system of a wind turbine. A gear box consists of gears, bearings and shafts that are subject to continual variable operational speed and loads. In a gearbox, the high-speed shaft is supported by the high-speed stage bearings located at the front and back end of the shaft. Typical operating speed of the shaft is between 1500 and 1800 rpm during power generation. It has been reported that a great number of wind turbine failures are related to the high-speed shaft bearings [2]. Cyclic loads caused by the wind turbine rotor blades will drive the main shaft to bend, leading to misalignment between the generator and the high-speed shaft and accordingly misalignment within the bearings. High-speed shaft bearings are therefore subject to damage from the cyclic loads [3,4]. For inspection purposes, wind turbine gearboxes are largely inaccessible since these are situated at the top of high towers. Possible failure implications are compounded by the fact that once a bearing fails, it may cause damage to the surrounding components of the gearbox, and as a consequence, cause replacement of various components inside the gearbox [5,6]. 

**Figure 1:**
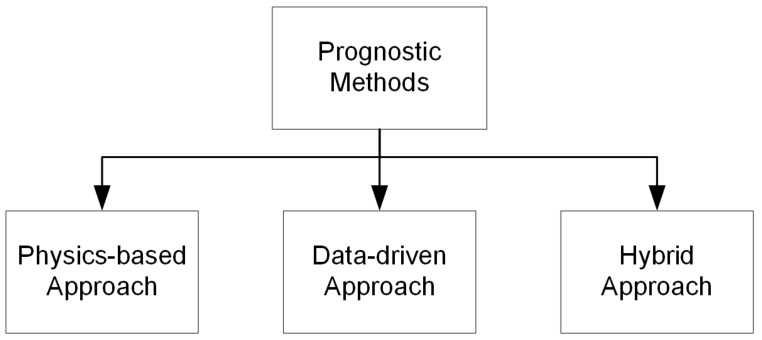
Main Prognostics Approaches.

Over the past decades, much research has been dedicated to the development of health monitoring methods for rotating machinery, especially the bearings. Compared to fault detection, the literature of prognostics and health management is relatively limited, and effective implementation of prognostic techniques is still lacking. The increased interest on machinery prognostics has resulted in many successful tools, models and applications during the past few years. Basically, there are three types of prognostic approaches that can be employed to predict the RUL, namely, data-driven methods, physics-based models and hybrid models (see Figure 1). Data-driven approaches utilize the historical failure data of the machine and/or similar machines to estimate how much time is left until a system malfunction occurs. This method does not require an in-depth understanding of the physics of system under study. Physics-based approaches predict the remaining life according to propagation of damage mechanism (i.e., physics of failure). A hybrid approach uses both data-driven and physics-based method so as to achieve an improved predictive performance in terms of improved predictive accuracy than when a single method is used.

Over recent years much efforts have been focused on developing regression-based prognostic methods that can be used to estimate the RUL of rotating machinery. Li et al. [7] improved the performance of traditional exponential regression model and applied the developed regression model to vibration measurements collected from rolling element bearings to predict RUL. Wu et al. [8] put forward a time-to-failure prognostic method based on empirical Bayesian algorithm an exponential regression model for rolling element bearings. Sutrisno et al. [9] investigated the accuracy of three different techniques for predicting the RUL of bearings. Bayesian Monte Carlo and moving average spectral kurtosis, support vector regression (SVR) and an anomaly detection algorithm were compared according to their performance in estimating a ball bearing’s remaining life. The anomaly detection technique was found to be the most accurate among all methods compared. Goebel et al. [10] conducted a comparative study of three prognostic methods RVM, Gaussian process regression (GPR) and a neural network. The study showed that the three techniques have resulted in significantly different RUL prediction results. Loukopoulos et al. [11] studied the performance of several machine learning techniques, including linear regression, polynomial regression and K-Nearest Neighbors Regression. The results showed that an ensemble method which is based on the weighted average of the predicted RUL of each individual method offers a higher predictive accuracy. Kim et al. [12] utilized Support Vector Regression to evaluate the bearing health condition by using real-world run-to-failure data obtained from bearings of gas pumps. The results showed that the developed probability estimation based prognostic method is potentially very effective for RUL prediction. A combined regression technique which is based on linear and quadratic regressions was put forward to deal with gas turbine engine’s degradation [13]. An e-support vectors regression model was proposed in [14] for the RUL estimation of rolling element bearings. The Logistic regression model was employed in [15] for the estimation of RUL of CNC machine. More regression-based prognostic models can be found in the literature [16,17,18,19,20].

Several other artificial intelligence approaches applied to machinery prognostics have been considered by researchers. For instance, a self-organizing neural network was employed by Zhang and Ganesan [21] for extrapolating the fault progression and estimating the remnant life of a bearing. Loukopoulos et al. [11] applied a Self-Organizing Map (SOM) model to predict the RUL of industrial pumps using temperature measurements. Dong et al. [22] developed a condition prediction method based on grey model and back-propagation neural network. Elasha et al. [23] put forward a life assessment approach for tidal turbine gearboxes. The method was validated on data generated using a Blade Element Momentum Theory (BEMT) model. They predicted the RUL of a gearbox based on the turbine loading conditions. The results of their investigation show life variations between the gears due to differences in stress cycles and differing rotational speeds. Li et al. [24] put forward a hybrid method in which a long short-term memory model and a state-space model was combined to predict the pro-fault performance of a centrifugal compressor. An adaptive neuro-fuzzy inference system (ANFIS) was used together with the particle filtering (PF) algorithm in [25] to predict the RUL of a gearbox. The authors concluded that the ANFIS model outperforms the recurrent neural network through a comparative study. Elforjani and Shanbr [26] employed three supervised machine learning techniques—artificial neural network (ANN), SVR and GPR—to correlate vibration measurement features with the natural wear of bearings. They concluded that the back-propagation neural network model outperforms the other methods in predicting the RUL of bearings. A prognostic framework based on auto-adaptive dynamical clustering was put forward by Chammas et al. [27]. This method allows the estimation of remnant life of incipient failure of a wind turbine benchmark. The RUL is estimated by using an auto-regressive integrated model to predict the future values of a severity indicator. A feed-forward neuro network was developed to learn the correlation between the lifetime and the health indicator extracted from the raw sensor signals [28]. Similarly, self-organizing map (SOM) and a feed-forward neural network were combined for effective bearing failure life prediction [29]. More prognostic methods based on feed-forward neural network can be found in [30,31,32]. Moreover, neuro-fuzzy systems whose membership functions are tuned by ANNs have also gain popularity in machinery prognostics. In [33], a multi-step forecasting model based on a weighted recurrent neuro-fuzzy system was put forward. A neuro-fuzzy system was utilized in combination with regression trees and particle filter in [34,35] for machine remaining useful life prediction. More research related to neuro-fuzzy based prognostics can be found in [36,37]. The aforementioned prognostic techniques offer a tradeoff between reliability, speed and applicability. Other techniques exit with wide ranging advantages and disadvantages [38], however this paper focuses on combining two supervised machine learning techniques, namely, regression model and artificial neural network (ANN) model to correlate vibration features with the corresponding fault stages during the natural run-to-failure process of rolling element bearings. One of the main contributions of this study is to improve the fitting of features obtained from vibration signals using appropriate regression models. This study also aims to ascertain the feasibility of using ANN models to estimate the RUL of rolling element bearings, and to explore the feasibility of combining regression models with ANNs for a better RUL prediction. The proposed combined technique leverages the strengths of both ANN and regression models, and is able to provide more accurate RUL estimations compared to traditional exponential regression models. Compared with traditional artificial intelligent methods, the proposed model takes advantages of the exponential regression approach in that the fitted prognostic features ensure precise modelling of the bearing degradation process. The effectiveness of the proposed prognostic method was validated on vibration measurements captured from an operational wind turbine gearbox.

## 2. Working Methodology

In condition monitoring applications, the vibration signals of bearing damage often present multiple modulation characteristics, and therefore the features extracted by the general methods from one bearing may not necessarily correlate to fault characteristics extracted from another bearing. The internal reasons behind this include, for example, different observed trends from different cases. As a result, there is still a need to apply and validate bearing fault indicators such as root mean square (RMS) and Kurtosis (KU) for different applications. Furthermore, due to the measurement noise, variation of operating conditions and stochasticity of the system deterioration, the extracted condition indicators from the raw vibration signals generally contain fluctuations, which would incur inaccurate RUL predictions. Thus, one of the main contributions of this study is to improve the fitting of the features extracted from vibration measurements through the use of appropriate regression models. This study also aims to ascertain the feasibility of ANN models to predict the RUL of rolling element bearings used in real-world applications, and to explore the possibility of combining regression models with ANNs to form a better prognostic model.

### 2.1. Statistical Condition Indicators

Vibration-based health monitoring schemes are applicable for monitoring many constituent components of a gearbox, such as shafts, gears and bearings. To achieve a better signal-to-noise ratio (SNR), vibration measurements are processed using filtering and amplifying techniques. Two condition indicators, RMS and KU, are often generated from the vibration signals. Then the extracted condition indicators are fitted using regression methods to provide useful information about the bearing degradation. There are several fault indicators (statistical features) that could be used for fault diagnostics using vibration data, such as RMS, KU, crest factor and energy operator, to name a few [39]. Among the aforementioned fault indicators, RMS and KU are the most widely used [40,41,42]. In this study, we compared the suitability of three different fault indicators (i.e., RMS, KU and energy index) for prognostic analysis. Monotonicity and trendability were utilized as the performance metrics for measuring the suitability of these indicators. Based on the obtained results, RMS and KU were eventually found to be most suitable indicators for the prediction of RUL in this study. 

#### 2.1.1. Kurtosis

Each mechanical failure has an associated “signature” that can be found in the frequency or time domain representations of vibration signals. Kurtosis is such a “signature” which is referred to as the fourth statistical moment of a given signal, reflecting the peakness of the histogram [43]. A kurtosis value greater than three is an indicator of a sharp peak signal. A kurtosis value smaller than three indicates vibration signals with flat peaks. In some cases, the occurrence of background noise and other sources of vibration signals may prevent bearing faults from being detected through the observation of changes in the kurtosis. To solve this problem, the kurtosis value needs to be computed across different frequency bands [44]. The Kurtosis of a random signal is computed as:

(1) $$KU=\frac{\frac{1}{N}\sum_{i=1}^{N}{\left(Xi-\mu \right)}^{4}}{{\left[\frac{1}{N}\sum_{i=1}^{N}{\left(Xi-\mu \right)}^{2}\right]}^{2}}$$

where N is the number of samples in the signal, Xi refers to the amplitude of the signal of the ith sample, and μ denotes the mean sample amplitude.

#### 2.1.2. Root Mean Square

The root mean square (RMS) value describes the energy content of a signal. RMS is one of the most common used statistical parameter that describe the change in the dynamic of the machine [45]. For the signal of sample size N, the RMS value is calculated using the equation below:

(2) $$RMS=\frac{\sqrt{X_{1}^{2}+X_{2}^{2}+\cdots X_{N}^{2}}}{N}$$

It is well known that the RMS value is a weak method to detect the failure at its early stage because of the small energy generated by the defect which makes a small difference to the value of RMS. However, RMS is capable of reflecting the increase of the vibration energy as the fault progresses [7]. Consequently, RMS is employed as the prediction indicator in this study. In other words, the RUL is predicted by extrapolating the trajectory of RMS values. 

#### 2.1.3. Energy Index

Energy Index (EI) is defined as the square of the ratio of the RMS value of a signal segment to the overall *RMS* value of the same signal. This technique has been effectively applied to detect incipient failure of bearings [46]. In practice, an EI value of one indicates non-transient type waveforms, whereas and an EI value larger than one is often associated with transient characteristics. EI can be computed using the following equation:

(3) $$EI\;=\;{\left(\;\frac{RMSsegment\;}{RMSoverall}\right)}^{2}$$

### 2.2. Definition of the Remaining Bearing Life

**Figure 2:**
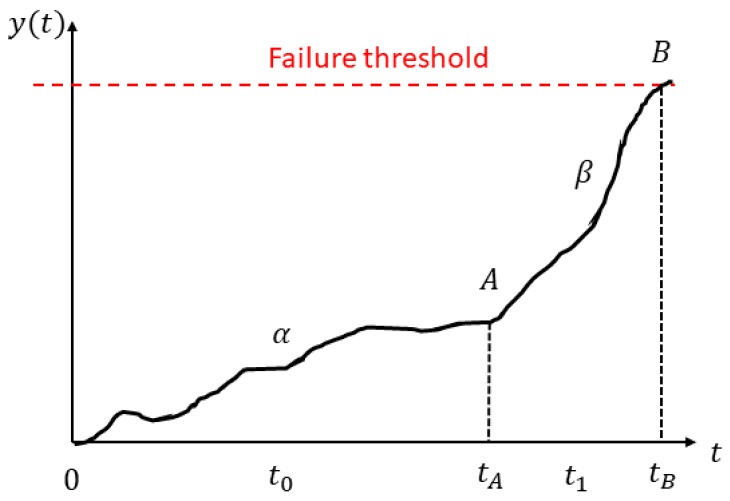
Bearing life process.

The RUL of a bearing is generally defined either as the total number of revolutions before a failure occurs or the total number of hours that the bearing can run until the first sign of failure develops [47]. The RUL is estimated based on measured and calculated bearing condition variables such as vibration amplitude and frequency. As shown in Figure 2, if a certain condition indicator x is calculated or monitored continuously from *t* = 0 to *t* = $t_{B}$, then a continuous time series *y*(*t*) can be obtained, which represents the deterioration process of the component under study. This time series consists of two parts, *α* and *β*, which indicates the healthy running stage and the fault degradation stage, respectively. Prognostic analysis is usually based on the analysis of the time series from point A to point B. Ideally, if the RUL of a bearing (i.e., the total running time between point A to B) can be accurately estimated by using only the past data covered by α, then the optimal maintenance schedule can be made easily.

### 2.3. Regression

Regression models, as one of the most popular data-driven techniques for RUL prediction, attempt to fit available data of deterioration by regression functions and then extrapolate the fault propagation until the fitted curve reaches a pre-defined threshold. The objective of regression analysis is to find an empirical relation for predicting the bearing degradation thought time series. Due to measurement noise, variation of operational conditions and the stochastic nature of the degradation processes, the acquired data are usually accompanied by fluctuations that may have a significant impact on the model’s ability to interpret the degradation trend. In this case, the raw condition indicators cannot be directly used as the inputs of the prediction models. This is due to the fact that any fluctuations in the condition indicators will cause the model to follow the randomness, and consequently, its ability to accurately estimate the health status of the bearings may be very weak [48]. Therefore, in this study, we first conduct a comparative study of two regression models, namely polynomial and exponential regression, and then choose the one with the best fitting performance to fit the condition indicators extracted from the data. 

The polynomial models are suitable for situations where the correlation between explanatory and study variables is curvilinear. Polynomial regression belongs to the least-squares curve fitting family. It takes a set of data as inputs and generate an approximation between the input data and time. To be specific, it estimates the coefficients of a polynomial function in that the function approximates the curve closely. The formula of polynomial regression is as follows:

(4) $$y=a_{0}+a_{1}x+a_{2}x^{2}+\cdots +a_{n}x_{i}^{n}$$

where *y* is the response variable, *x* is the predictor variable, and $a_{0}$, $a_{1}$, …, $a_{n}$ are model coefficients. The degree of the polynomial function is determined by the number of non-zero coefficients in Equation (4), which in turn determines how accurate the data can be fit. If the number of coefficients is one or two, then the fitted curve is known as a linear regression. If the number of coefficients is larger than two, a non-linear polynomial regression will be implemented.

**Figure 3:**
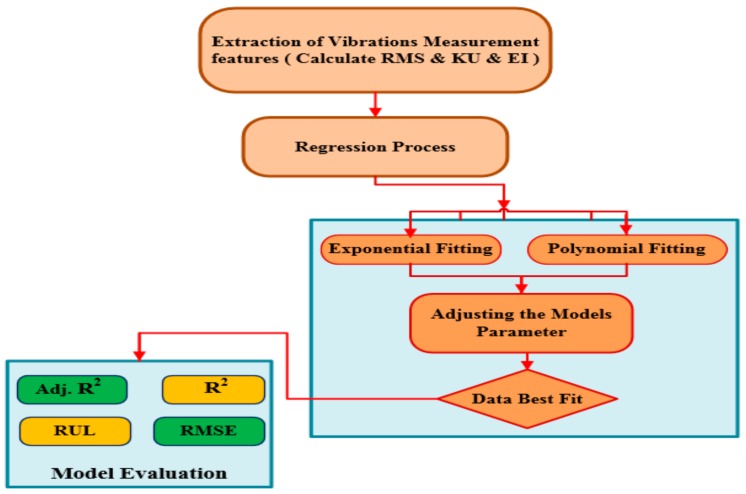
Schematic of regression process.

The exponential regression model is a fitting process that finds the equation of the exponential function which can present the best fit for a set of data [48]. The function form of the exponential is shown in Equation (5):

(5) $$y=a*\;e^{bx}$$

where a, *b* are model constants and ***x*** is the predictor variable. Figure 3 shows the process of two regression models that were used to extract the best fit from the scatters statistical parameters. In which the vibration signals have been processed to obtain the condition indicators. And then the exponential and polynomial functions have been used to determine the best fit and estimate the coefficients in Equations (4) and (5).

The performance of the regression models was assessed using three statistics: Root Mean Square Error (RMSE), R^2^ and Adjusted R^2^. The RMSE is the square root of the variance of the residuals. RMSE measure how close the measured data to the predicted values. The R^2^ is defined as the ratio between the difference between the sum square total (SST) and sum square error (SSE) to the SST. SST measures the data deviation from the sample mean, and SSE measures the deviation of the data from the model’s predicted values. Therefore, the R^2^ value provides the goodness of the data. One of the disadvantages of the R^2^ is that it can increase if there are more than one predictor, but this increase does not reflect the model improvement. Therefore, adjusted R^2^ has been used in this research. Adjusted R^2^ is defined as the ratio between the residual mean square errors to the total mean square error (which is the variance of the predicted values). 

#### Indicator Performance Quantification

Identification of a suitable indicator simplifies the degradation assessment and prognostics. Parameter features include monotonicity and trendability can be used to compare candidate prognostic parameters to determine which parameter is most useful for prognosis task [29]. Monotonicity and trendability are a type of metrics used to quantify the indicators suitableness and are defined as the following.

a) Monotonicity

The monotonicity metric indicates the principal whether or not the sequence is increasing or decreasing (positive or negative trend of the indicator). Due to the fact that the bearing degradation is considered to be an irreversible process, monotonicity measures whether or not a condition indicator is suitable for representing a degradation process. Monotonicity is calculated as per Equation (6):

(6) $$Monotonicity=mean\left(\left|\frac{\#pos\frac{d}{dx}}{n-1}-\frac{\#neg\frac{d}{dx}}{n-1}\right|\right)$$

where n denotes the number of measurement time instances. #$pos\frac{d}{dx}$ denotes the number of positive derivatives, and #$neg\frac{d}{dx}$ is the number of negatives derivatives.

The monotonicity of a sensor population is calculated by the average difference of the fraction of positive and negative derivatives for each path. A monotonicity value close to one means that the condition indicator is monotonic and suitable for RUL prediction, whereas a monotonicity value close to zero indicates that the condition indicator is non-monotonic and not appropriate for RUL prediciton. 

b) Trendability

The trendability metric indicates the degree to which the condition indicator values at different times have the same fundamental shape and can be defined using similar function form. Its value is determined by the minimum absolute correlation calculated among all the condition indicators [18]. A condition indicator can be considered trendable if all the parameters can be modelled by the same function (Equation (7)):

(7) $$Trendability=min\left(\left|corrcoeff_{ij}(x_{i},\;x_{j}\right|\right)\;i=1,2,\ldots ,n\;and\;j=1,2,\ldots$$

### 2.4. Multilayer Artificial Neural Network

ANNs belong to the supervised machine learning family. They are inspired by biological neural networks and each neuron is represented by a node [28]. An ANN generally contains an input layer, multiple hidden layers, one output layer, biases and connection nodes. When the known inputs and target outputs are repetitively presented to an ANN, the connection weights between nodes will be adjusted automatically such that the difference between the network outputs and the targets is as small as possible.

**Figure 4:**
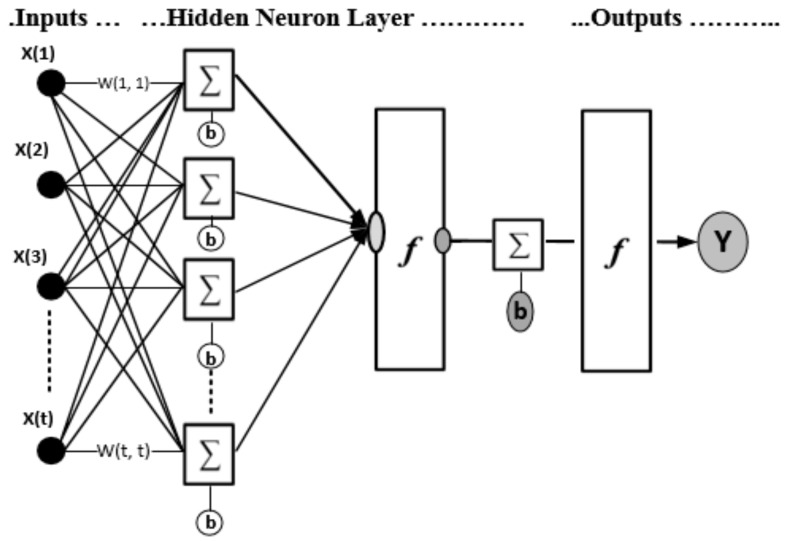
Multiple-layer neural network.

In this study, the ANN that we implemented is a multilayer back-propagation neural network. Figure 4 illustrates an exemplary architecture of the multiple-layer neural network model. It is observable from the figure that each layer consists of its own inputs and outputs nodes (x & y), weighting coefficients (**w**) and bias (**b**). The input layer doesn’t involve any processing and it is utilized to directly feed information to the subsequent network layers. In contrast, the output layer involves weighting and biases calculations and is employed to produce the network outputs. The hidden layers aim at adding additional processing to the network so as to avoid solutions that do not converge. As shown in Figure 4, the main purpose of the bias neurons is to prevent the network from generating zero results even if the network inputs are not zero. The exemplary network structure is formed of a feed-forward model. The following equation explains how the network inputs are correlated with the outputs:

(8) $$y_{i}=\varphi_{0\;}\left[C\varphi_{h}\left(Bu_{i}+b_{h}\right)+\;b_{0}\right]$$

where $y_{i}$ is the network output vector and the input vector is represented by $u_{i}$, **C** denotes the weighting matrix between the hidden layer and the output layer. ***B*** is the connection matrix from the input layer to the hidden layer. The bias vectors of the hidden and output layers are represented by $b_{h}$ and $b_{0}$, respectively. $\varphi_{h}$ and $\varphi_{0}$ denote the activation functions of the nodes in the hidden and output layers, respectively. Feedforward neural network models also take the form of Equation (9):

(9) $$y_{i}=f\left(u\right)$$

where f(·) denotes a nonlinear transformation from u to $y_{i}$. Interestingly, the structure of a feedforward neural network is similar to that of a nonlinear regression model. Levenberg Marquardt (LM) learning algorithm was chosen as the network training function in this study for adjusting the weighting and bias matrices during the training process. LM optimization has been applied intensively for feedforward neural network training and has been proven to be able to deal with many difficult and diverse problems in practice. This algorithm minimizes functions that are sums of squares of nonlinear functions. One of the advantages of this optimization method is that the second-order convergence point can be approached without calculating the Hessian matrix.

## 3. Data Collection

The vibration dataset was collected using the Green Power Monitoring System, and interested readers are referred to [49]. The data were collected from a high-speed shaft bearing mounted inside a 2 MW wind turbine. The vibration measurement was taken for 50 consecutive days using MEMS-based accelerometers mounted radially on the bearing support ring [49]. Data were collected at 10-minute intervals and the bearing speed was 1800 rpm. A total number of 50 data sets were recorded for analysis. The vibration data were sampled at a sampling rate of 97,656 Hz for 6 s [49]. Table 1 lists the key parameters of interest.

## 4. Results

### 4.1. Signal Features Regression

It has been mentioned previously that due to measurement noise, variation of operational conditions and the stochastic nature of the degradation processes, the raw condition indicators cannot be directly used as the inputs of the prediction models. To solve this problem, the obtained raw data was fitted by means of appropriate mathematical functions (i.e., exponential and polynomial models were utilized to represent trends of the condition indicators). 

**Figure 5:**
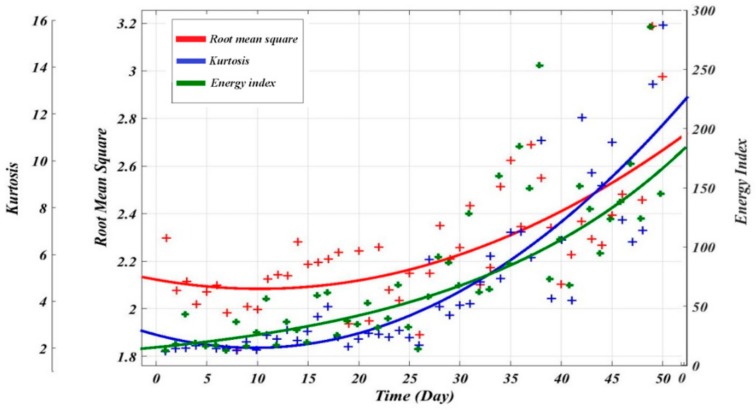
Fitted condition indicators using exponential functions.

**Figure 6:**
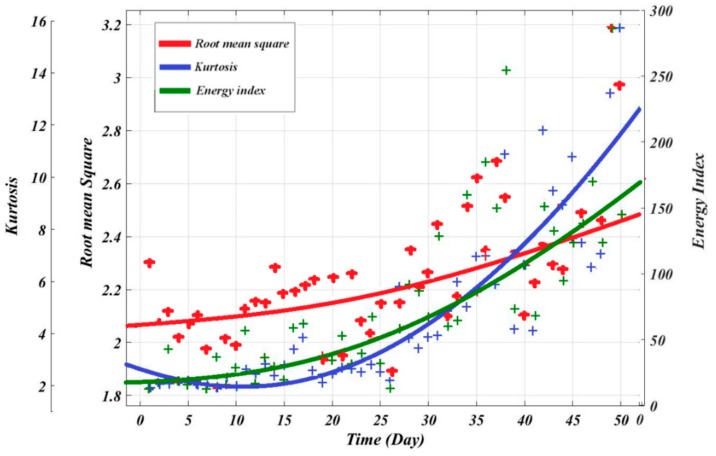
Fitted condition indicators using polynomial functions.

The regression model utilized the two equations mentioned above (i.e., Equations (4) and (5)) to find the relationship between condition indicators and time. Figure 5 and Figure 6 illustrate the actual and fitted condition indicators for 50 consecutive days of measurements of RMS, KU and EI. Table 2 and Table 3 also summarize the optimal model constants and the RMSE, R^2^ and adjusted R^2^ for the exponential and polynomial models respectively. It can be observed that using the exponential regression to fit the condition indicators is more accurate than the polynomial in this case study, see Table 2 and Table 3. For instance, its results for RMS showed the lowest value of root mean square error of 0.05248 and a high adjusted R^2^ with 0.957. Whereas, the results obtained from polynomial for the same condition indicator showed the lowest adjusted R^2^ with 0.5774. 

It is worth mentioning that a single metric only indicates the good fit to the data from one prospective, and does not necessarily indicate that the model parameters are individually well-determined. Therefore, three good to fit metrics were utilized in this study. In this work, several exponential and linear functions were applied to the raw data. As a final result, the following exponential and polynomial models have been utilized to fit the different fault indicators (see Table 2 and Table 3).

Observations of Table 2 and Table 3 showed that R^2^ and adjusted R^2^ values of the condition indicators are similar for all exponential models, however, the lowest RMSE was observed for the RMS. Comparison of the exponential models to the polynomial model showed the exponential model has the best performance based on all three assessing parameters.

**Figure 7:**
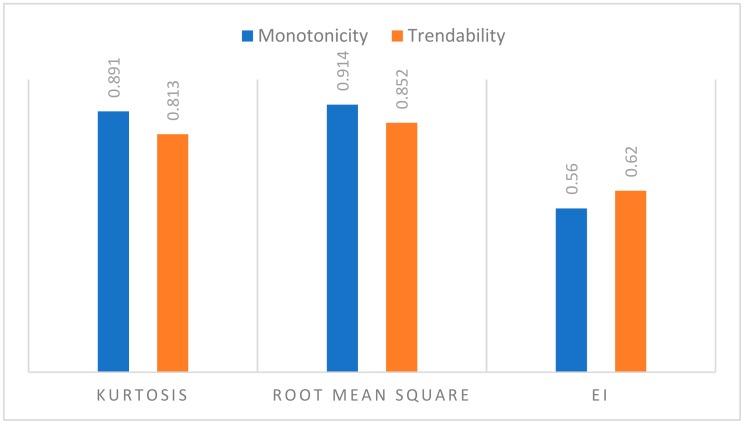
Evaluation of the condition indicators.

Figure 7 presents the evaluation of condition indicators; the results showed the RMS trendability and monotonicity parameters are higher compared to Kurtosis. Considering the values of the three metrics RMSE, monotonicity and trendability, the RMS is the best indicator for the representation of the degradation process compared with EI and KU. Therefore, it has been used for the ANN model as the best fit parameter (i.e., fitted RMS values are used for ANN outputs).

### 4.2. Neural Network

In this study, we propose to use an ANN model for estimating the RUL for bearing running at high speed and constant load. The model is a feed-forward back-propagation artificial neural network model with one input layer, a changeable number of hidden layers and one output layer. the optimal number of hidden layers, learning rate and algorithm type were determined during the training process by minimizing the error between the target outputs and the model outputs. Based on the training results, the ANN model that was finally adopted consists of one input layer with two inputs, namely raw RMS and KU; one output layer with one output (fitted RMS values); and two hidden layers with nine neurons in the first layer and seven neurons in the second layer. 

The Levenberg-Marquardt (LM) algorithm was utilized to train the model. LM has been proven to outperform traditional gradient descent and many conjugate gradient-based algorithms in many applications [50]. LM has the local search properties of the Gauss–Newton algorithm, but with consistent error decrease thanks to the gradient descent algorithm. It updates the network weighting and bias matrices according to LM optimization. The regularization diminishes a compound of squared errors and weights to reduce the computational overhead.

**Figure 8:**
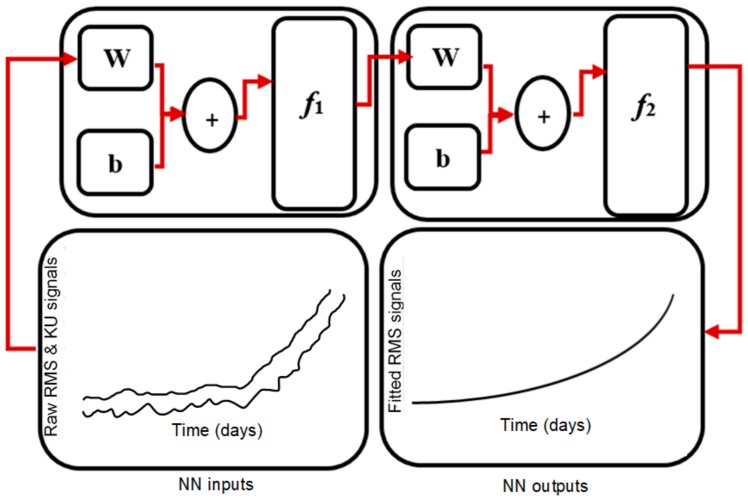
Neural network training stage.

Figure 8 shows the configuration of the training stage. The inputs of the ANN model are the raw RMS and KU values, while the target is the best fitted RMS obtained using fitting tools for the same parameter settings. 

The network structure maps between these variables to generate the function of explaining their relationship. The extracted raw RMS and KU were fed to the model, and the model was trained using the algorithms stated previously. As mentioned previously, the lowest training errors were obtained by an ANN model containing one input layer, one output layer and two hidden layers. The layer size was determined according to the mean square errors between the target output series and the estimated outputs. 

**Figure 9:**
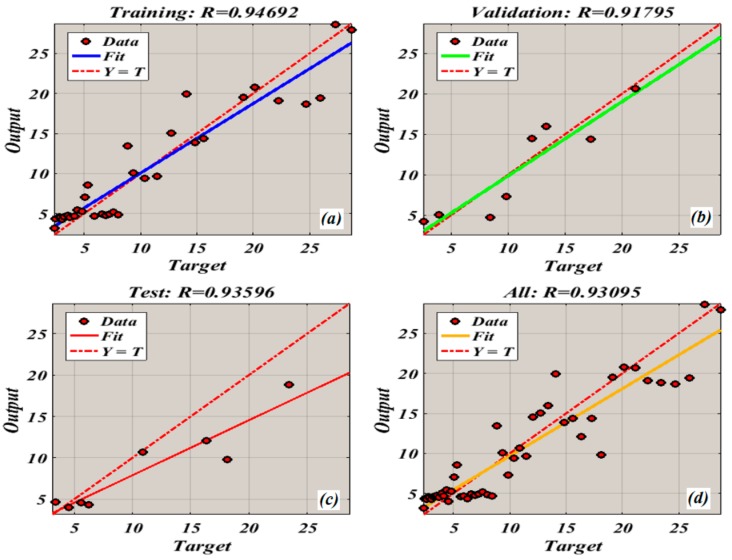
Neural Network Training Regression: (**a**) training results.; (**b**) validation results.; (**c**) test results.; (**d**) results obtained using all data.

The next phase comprised of validation of the trained model. This was achieved by passing the raw KU and RMS values to the trained model. The ANN model’s output (i.e., estimated fitted RMS) is then extrapolated to a pre-defined failure threshold to predict the RUL of the system. Figure 9 presented the final results of model output obtained from 70% of training and 30% of testing data. The error and the RUL were calculated using the following equation:

(10) $$RUL=t_{f}-t_{c}$$

where $t_{f}$ is the time at which the actual failure occurred; in this study, $t_{f}$ was chosen as the last time instance of running, which means the bearings had a damage at this point and then stopped. The $t_{c}$ is the current time. The RUL calculated based on Equation (10) is the actual RUL. The estimated RUL is calculated based on the same $t_{c}$ and the $t_{f}$ obtained by extrapolation for pre-defined threshold. 

The prediction errors are calculated based on the difference between the actual and the estimated RULs:

(11) $$Error\;\left(\%\right)=\frac{Actual\;\left(RUL\right)-Estimated\;\left(RUL\right)}{Actual\;\left(RUL\right)}$$

(12) $$Sum\;Square\;Error=\frac{1}{2}\overset{n}{\underset{i=1}{\sum }}Error_{i}^{2}$$

**Figure 10:**
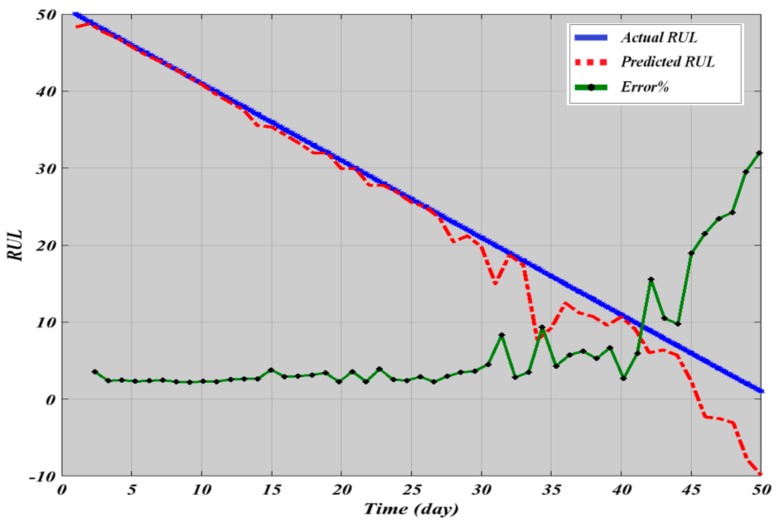
Regression Model RUL results.

**Figure 11:**
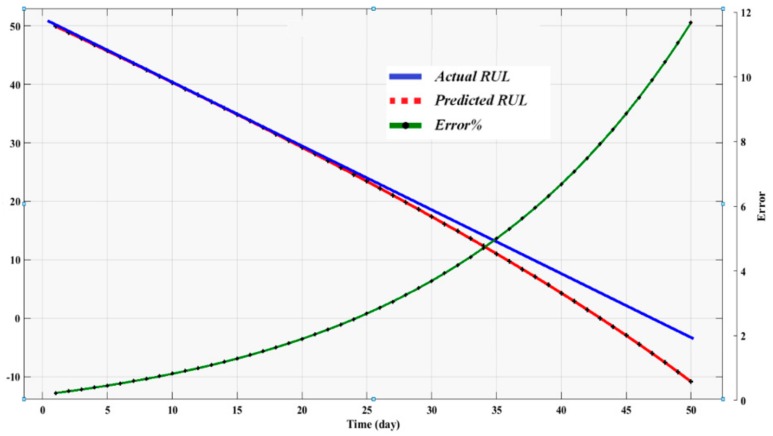
Artificial neural network RUL results.

The sum of the square error resulted from regression and ANN are presented in Table 4. Observation of the results show that the lowest error SSE are registered by the ANN. Interestingly some negative error values were recorded due to the fact the models have overestimated the RUL. Based on observation of the trends of the estimated RUL compared to the actual RUL (Figure 10 and Figure 11), it is evident that the proposed approach follows satisfactorily the deterioration trend of the bearings under study with small errors. Figure 10 depicts the estimated RUL with the actual bearing life using the exponential regression model as a prediction tool. The raw RMS was fitted using an exponential regression model as stated in Table 2. We then extrapolate the time at which the fitted RMS exceeds a pre-defined threshold to obtain the predicted failure time. It shows that the trend at the beginning of the prediction initially closely estimated the actual values. However, the prediction deviates from the actual values and oscillates away as the actual end-of-failure approaches. At this stage the fault can be detected by the condition monitoring system, and thus there is no need for prognosis to predict the fault stage. Results obtained from ANN analysis using two inputs (RMS and KU) are depicted in Figure 11. For the ANN case, we simulate the fitted RMS as the ANN outputs, and then the simulated RMS is extrapolated to a pre-defined threshold to predict the RUL. 

The mean square error (MSE) is a network performance function that measures the network’s performance according to the mean of square errors. When the vector of predictions is obtained and the vector of known true RUL values is available, MSE can be estimated by:

(13) $$MSE=\frac{1}{n}\overset{n}{\underset{i=1}{\sum }}{\left(y_{i}-{\hat{y}}_{i}\right)}^{2}=\frac{1}{n}\overset{n}{\underset{i=1}{\sum }}e_{i}{}^{2\;}$$

where ${\hat{y}}_{i}$ is the estimated value (ETTF) and $y_{i}$ is the desired output value (ATTF).

Table 5 shows the MSE for both regression and ANN models. Results observation shows that the prediction made by the proposed model is very accurate. In addition, the best performance of the ANN model has been recorded at an MSE of almost 5.62 which is a good indicator of the model accuracy.

## 5. Conclusions

A data-driven prognostic method has been developed and tested using vibration signals collected from an operational wind turbine gearbox. This paper has addressed bearing prognosis with the aim of predicting the RUL of high-speed shaft bearings. Two types of prediction methods, namely, regression and back-propagation neural network, have been used to model and estimate the remnant life. The regression model’s results have been used to feed the neural network to enable better predictions. 

The proposed model was tested on real-world vibration data collected from a 2 MW wind turbine (degradation of bearing operating at speed of 1800 rpm). The obtained results using regression and ANN models have been compared. The regression was based on three condition indicators RMS, Kurtosis, and EI. The performance of each regression model was compared using three parameters: RMSE, R^2^ and adjusted-R^2^. The result showed that the exponential model has the best performance. The ability of condition indicators to be used for prognosis has been evaluated using monotonicity and trendability parameters. The results showed RMS has the best overall performance, therefore the RMS was used as best fit output data for neural network. 

The results obtained using the proposed ANN model indicate that it has good performance in predicting the remaining useful life of a bearing, and this success can be attributed to the link created between the regression model to the ANN through the best fit condition indicator. Comparing the performance of regression model and the ANN it can be seen that the ANN model was able to provide more accurate predictions, however, this performance cannot be achieved without the regression model. Therefore, the regression model is considered necessary to improve the predictive performance of the ANN model. The stochastic nature of the degradation processes cannot be mitigated by just fitting a regression model. The use of a probabilistic model can overcome this limitation. Efforts will be made in future research to explore the RUL prediction using a probabilistic approach.

## Figures and Tables

**Table 1:** Wind turbine operating details.

Machine State	Increasing Inner Race Bearing Fault
Power rating	2 MW flux
Nominal speed	1800 rpm
Measurement Channel	Sensor
Sample rate	97656 Hz
Record length	6 s
Sensor type	Accelerometer

**Table 2:** General optimal estimated exponential model constants.

Condition Indicators	Model Constants		RMSE	R^2^	Adj. R^2^
	a	b			
RMS	2.235	0.0511	0.0525	0.958	0.957
KU	3.439	0.112	0.947	0.977	0.975
EI	54.07	0.634	9.711	0.968	0.967

**Table 3:** General optimal estimated polynomial model constants.

Condition Indicators	Model Constants			RMSE	R^2^	Adj. R^2^
	a_0_	a_1_	a_2_			
RMS	2.19	0.117	0.0044	0.164	0.595	0.577
KU	3.24	0.572	0.3004	0.217	0.881	0.875
EI	53.8	38.81	11.2	13.94	0.945	0.942

**Table 4:** Sum Square Error Results.

Model	SSE
Polynomial	8427
Exponential	5419
ANN	661.198

**Table 5:** Error results.

Model	MSE
Polynomial	15.61
Exponential	12.56
ANN	5.62
